# Differences in Messenger RNA Expression of Fibulin-1, Elastin, Matrix Metalloproteinase-1, Basic Fibroblast Growth Factor, and α-Smooth Muscle Actin Between the Ventral and Dorsal Tunica Dartos in Patients With Hypospadias and Chordee: Protocol for a Prospective Cohort Study

**DOI:** 10.2196/52282

**Published:** 2024-10-30

**Authors:** Joko Pitoyo, Alvarino, Eryati Darwin, Yanwirasti

**Affiliations:** 1 Faculty of Medicine Universitas Andalas Padang Indonesia; 2 Faculty of Medicine Riau University - Arifin Achmad General Hospital Riau Indonesia

**Keywords:** chordee, gene expression, hypospadias, polymerase chain reaction, mRNA

## Abstract

**Background:**

Hypospadias is a common congenital anomaly characterized by the displacement of the urethral opening to the ventral side of the penis. Surgical correction is often necessary for functional and psychological reasons. The etiology involves genetic and environmental factors, and chordee, a downward curvature of the penis, is a common complication. Proteins such as fibulin-1, elastin, matrix metalloproteinase-1, basic fibroblast growth factor, and α-smooth muscle actin play roles in hypospadias development.

**Objective:**

The study’s aim is to investigate the differences in messenger RNA (mRNA) expression of fibulin-1, elastin, matrix metalloproteinase-1, basic fibroblast growth factor, and α-smooth muscle actin between the ventral and dorsal tunica dartos in patients with hypospadias and chordee.

**Methods:**

This prospective cohort study aims to investigate differences in mRNA expression of the abovementioned proteins between the ventral and dorsal tunica dartos in patients with hypospadias and chordee. Ethics approval has been obtained, and consent from parents will be obtained before data collection. Eligible participants are aged 6-18 months, diagnosed with hypospadias and chordee, and planned for urethroplasty. Tissue samples will be collected from both aspects of the tunica dartos and analyzed using real-time quantitative reverse transcription–polymerase chain reaction. Data analysis will involve statistical tests and normalization of housekeeping genes.

**Results:**

This study is at the protocol development stage. A pilot study regarding its feasibility has been ongoing as of August 2023. The study results are expected to be available by the end of 2024.

**Conclusions:**

The study of mRNA expressions of various proteins in the tunica dartos of patients with hypospadias and chordee is expected to improve the understanding and expand the knowledge of the pathophysiology of hypospadias and chordee.

**International Registered Report Identifier (IRRID):**

DERR1-10.2196/52282

## Introduction

Hypospadias is one of the most common congenital anomalies in male neonates. It is characterized by the urethral opening being misplaced on the ventral side of the penis instead of the tip. Its prevalence globally is around 1 in 200-300 male births [1]. It has significant psychological and functional impacts, requiring surgical correction to achieve normal appearance and function. Various surgical techniques exist, but controversies persist regarding the best approach for different types of hypospadias [2]. The etiology of hypospadias is believed to be multifactorial, involving genetic and environmental factors [3,4]. Chordee, a downward curvature of the penis, is commonly associated with hypospadias and results from tissue growth disturbances [5].

The proteins fibulin-1, elastin, matrix metalloproteinase-1 (MMP-1), basic fibroblast growth factor (bFGF or FGF-2), and α-smooth muscle actin (α-SMA) play roles in hypospadias development [4,6,7]. Fibulin-1 is related to extracellular matrix integrity, while elastin contributes to tissue extensibility. MMP-1 is involved in matrix remodeling, while bFGF affects differentiation. α-SMA is implicated in fibrosis. Messenger RNA (mRNA) expression of these proteins may shed light on their roles in hypospadias [2,4,8].

However, research on dartos tunic elasticity, genetic factors, and mRNA expression in hypospadias is limited. A better understanding of the genes influencing dartos tunic elasticity could provide insights into pathophysiology and chordee management. Therefore, this protocol is designed to investigate the differences in mRNA expression of fibulin-1, elastin, MMP-1, bFGF, and α-SMA between the ventral and dorsal tunica dartos in patients with hypospadias and chordee.

## Methods

### Study Design

This is an observational study using a prospective cohort, designed for the investigation of the differences in mRNA expression of fibulin-1, elastin, MMP-1, bFGF, and α-SMA between the ventral and dorsal tunica dartos in patients with hypospadias and chordee. The protocol is prepared according to the SPIRIT (Standard Protocol Items: Recommendations for Interventional Trials) 2013 checklist for reporting a protocol study [9].

### Ethical Considerations

This study was conducted in accordance with the principles outlined in the Declaration of Helsinki. The approval for the protocol of this study was granted by the Medical Research Ethics Committee, Universitas Indonesia, in April 2022 (KET-413/UN2.F1/ETIK/PPM.00.02/2022). All participants provided written informed consent prior to their inclusion in the study, and the study protocol was reviewed and approved by the institutional review board to ensure ethical standards and participant safety.

Informed consent was obtained from all participants during the primary data collection phase. The original informed consent covered the possibility of secondary data analysis, and the institutional review board confirmed that no additional consent was required for this study’s secondary analysis.

To ensure the privacy and confidentiality of participants, all study data were deidentified prior to analysis. Personal identifiers were removed, and the data were anonymized to protect the participants’ identities. Appropriate measures were taken to maintain the confidentiality of the data throughout the study.

No additional compensation was provided for this analysis as it did not involve additional participant interaction.

There are no images included in the manuscript or supplementary material that could potentially identify individual participants. If such images are deemed necessary in future submissions, we will ensure that consent has been obtained from identifiable individuals, and relevant consent forms will be uploaded during resubmission.

Consent for the data collection and publication has been obtained from the parents of patients with hypospadias. The consent was sought after the parents and their children have already received an explanation from the main researcher.

The data taken for this study were stored and uploaded over the internet without any identifier.

### Eligibility Criteria and Recruitment Procedures

The eligibility criteria for participants in this study are as follows:

- Inclusion criteria:
  1. Diagnosed as having hypospadias with chordee by a pediatric urologist
  2. Aged 6-18 months
  3. Planned to undergo urethroplasty
- Exclusion criteria:
  1. The amount of available tissue specimens is insufficient for further examination
  2. Patients with genital ambiguity characterized by clitoral hypertrophy
  3. The patient is experiencing an infection of the prepuce
  4. Patients with a history of previous chordectomy or urethroplasty surgery
  5. Patients with conditions that can experience dysregulation of extracellular matrix component proteins, namely malignancy and Marfan syndrome

Participants for this research will be enrolled through a consecutive recruitment method. Parents of eligible patients will receive information about the study’s procedures and will be given the opportunity to decide whether they wish for their children to take part in the study during its duration.

### Clinical Outcomes

The clinical outcomes measured in this study are as follows:

1. Expression of fibulin-1 mRNA
2. Expression of elastin mRNA
3. Expression of MMP-1 mRNA
4. Expression of bFGF mRNA
5. Expression of α-SMA mRNA

The clinical outcomes will be measured from differences in mRNA expression from both the dorsal and ventral aspects of the tunica dartos excised during urethroplasty. A positive control gene for both dorsal and ventral tissues will also be measured in subsequent analyses. The expression levels of the 5 genes will be assessed relative to the housekeeping gene β-actin using fold change values.

### Randomization and Blinding

This study is single blinded. The laboratory assistant does not know which aspect of the penis that the specimen belongs to.

### Study Process and Data Collection

#### Specimen Collection

**Figure 1:**
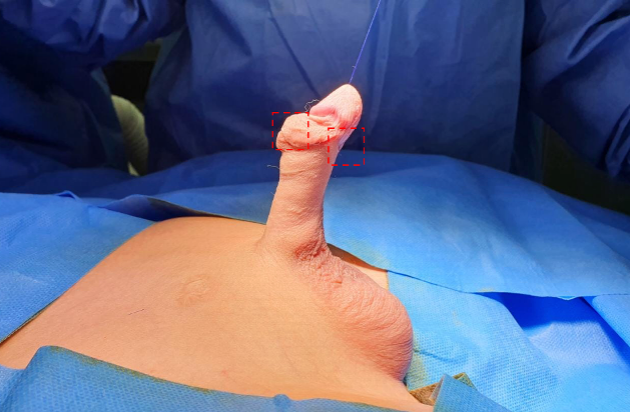
Site of tissue sample collection from the dorsal and ventral sides of the tunica dartos from patients with hypospadias and chordee, from an observational study conducted in Arifin Achmad General Hospital Riau in 2023 (source: personal documentation of the researcher).

In this study, tissue samples from the dorsal and ventral sides of the tunica dartos will be obtained from patients with hypospadias and chordee. Ventral tunica dartos samples will be taken from each patient by the researcher as the performing operator, ensuring that the size and location of collection are standardized across all patients. The collected portions are shown in Figure 1.

#### Total RNA Isolation

RNA will be isolated from all samples using the RNeasy Mini Kit (Qiagen) following the kit’s manual instructions. Initially, β-mercaptoethanol and ethanol are added to Buffer RPE. Tissue samples are separated from RNAlater stabilization and then mixed with Buffer RLT. For tissue weighing 20-30 mg, 600 µL of Buffer RLT is added. Sample disruption and homogenization are performed using an ultrasonic disruptor tool for 20-40 seconds. The lysed samples are centrifuged at 17,000×g for 3 minutes, and the supernatant is collected for RNA extraction. Then, 70% ethanol, equal to 1× the lysate volume, is added and homogenized using a micropipette.

A total of 700 µL of the sample is transferred to a QIAamp spin column placed in a 2-mL collection tube and centrifuged at >8000×g for 1 minute. The supernatant is discarded, and the process is repeated by pairing a new 2-mL collection tube with the QIAamp spin column until all lysates, including precipitates, are processed. The supernatant and 2-mL collection tube are discarded. The QIAamp spin column is then transferred to a new 2-mL collection tube.

Buffer RW1 (700 µL) is added to the QIAamp spin column and centrifuged at >8000×g for 1 minute. The supernatant and 2-mL collection tube are discarded. This step is repeated with the addition of 500 µL of Buffer RPE. The QIAamp spin column is then combined with 500 µL of Buffer RPE and centrifuged at 17,000×g for 3 minutes, and the supernatant is transferred to a new 2-mL tube and centrifuged at 17,000×g for 1 minute. The QIAamp spin column is moved to a new 1.5-mL microcentrifuge tube, and 30-50 µL of RNase-free water is added directly to the QIAamp membrane, followed by centrifugation at >8000×g for 1 minute.

#### Quantification of RNA and Circular DNA Synthesis

The quantification stage serves to determine the RNA concentration in each sample in order to dilute them to the same concentration, which is 0.2 ng/µL. RNA quantification from each sample and control will be performed using the Qubit dsDNA HS kit (Q32851; Thermo Fisher Scientific). All samples from the whole genome amplification stage are vortexed and diluted by mixing 5 µL of the sample with 45 µL of double-distilled water to achieve a 1:10 dilution. Samples, controls, and the Qubit standard solution are placed in 500-µL tubes containing Qubit dsDNA reagent with specific volumes.

All samples are then vortexed, spun down, and incubated at room temperature for 2 minutes. The RNA quantification results are then used for circular DNA synthesis using the cDNA Taqman MicroRNA Reverse Transcription Kit (4366596; Thermo Fisher Scientific). The composition of the reagents used can be seen in Hidayati et al [10]. The master mix, containing the samples, is then incubated in a thermal cycler.

#### Expression Quantification Using a Real-Time Quantitative Reverse Transcription–Polymerase Chain Reaction Machine

A real-time quantitative reverse transcription–polymerase chain reaction (qRT-PCR) machine will be used to perform the qRT-PCR amplification using the primary sequences in Table 1.

The machine monitors fluorescence levels after each PCR cycle to track the amount of amplified DNA in real time.

**Table 1:** Primary sequences of the genes used in the study.

Gene	Nucleotide code	Primary sequence (5’-3’)
Fibulin-1	NM_006486.3	F^a^: GATGGTGTCTCCTGTGAAGATG R^b^: TGATGCATGTATGCCCGATAG
Elastin	NM_000501.4	F: CTGCAAAGGCAGCCAAATAC R: CACCAGGAACTAACCCAAACT
MMP-1^c^	NM_002421.4	F: GCCTTCCAACTCTGGAGTAATG R: GAATGGGAGAGTCCAAGAGAATG
bFGF^d^	NM_001361665.2	F: TGAAACGAACTGGGCAGTATAA R: TTGACCTGACTGTGGAAGAAC
αSMA^e^	NM_001613.4	F: GACCCTGAAGTACCCGATAGA R: CTCAGCAGTAGTAACGAAGGAATAG

^a^F: forward.

^b^R: reverse.

^c^MMP-1: matrix metalloproteinase-1.

^d^bFGF: basic fibroblast growth factor.

^e^α-SMA: α-smooth muscle actin.

#### Data Analysis

The amplification curve data will then be analyzed to determine the cycle threshold (Ct) value. The Ct values are then normalized to that of the housekeeping gene to calculate the relative gene expression using the ΔΔCt method.

### Data Analysis

The data to be collected in this study include clinical characteristics such as age, height, weight, BMI, comorbidities, and delivery history, accompanied by mRNA expression from both the dorsal and ventral sides of the tunica dartos tissue.

All quantitative data obtained from this research will be analyzed using SPSS (version 25.0; IBM Corp) software. The normality of all numeric data will be tested using the Kolmogorov-Smirnov test. Numeric data will be presented as mean (SD) for normally distributed data and median (range) for nonnormally distributed data.

If the obtained data are normally distributed, an independent 2-tailed *t* test will be performed. Meanwhile, nonnormally distributed data will be tested using the Mann-Whitney *U* test to determine gene expression differences between research groups. The quantification of gene expression is expressed as fold change, compared to the housekeeping gene used in the study.

## Results

This study is at the protocol development stage. A pilot study regarding its feasibility has been ongoing as of August 2023. The study results are expected to be available by the end of 2024.

## Discussion

Hypospadias, characterized by the abnormal positioning of the urethral opening, is a prevalent condition that ranks as the second most frequent congenital anomaly in male neonates, following cryptorchidism. The condition is typified by the displacement of the urethral meatus to the ventral side of the penis, leading to curvature abnormalities in the penis and preputial skin. The quest for optimal restoration, both functionally and aesthetically, drives the need for hypospadias correction surgeries. The primary aim of these interventions is to achieve acceptability in both the appearance and function of the penis, while also addressing psychological concerns [1,2].

The realm of hypospadias surgery presents a diversity of approaches for penile reconstruction, yet controversies persist regarding the most suitable techniques for various types of hypospadias and the utilization or excision of tunica dartos in surgical procedures. Noteworthy among these techniques is the “tubularized incised plate” urethroplasty proposed by Snodgrass et al [11], utilizing tunica dartos excision to address chordee. Another technique, similar in approach but employing tunica dartos or tunica vaginalis as a second layer, was presented by Tavakkoli Tabassi and Mohammadi [12]. Diverging opinions among urologists surround the excision of all inelastic tissue (ventral tunica dartos), coupled with dorsal tunica dartos as a second layer. Intriguingly, prior studies failed to find disparities between the ventral and dorsal tunica dartos, attributing this to their concurrent formation and common origin [4,7,13].

The multifactorial etiology of hypospadias is underscored by its potential maternal and paternal inheritance, suggesting a likelihood of 57% to 77% heritability. Environmental factors, including exposure to chemicals, such as pesticides, diethylstilbestrol, vinclozolin, polychlorinated biphenyls, phthalates, and dioxins during pregnancy, have been implicated in hypospadias causation [3,4]. While the exact etiology of hypospadias remains elusive, histopathological findings in patients with hypospadias warrant further investigation into the disorder’s etiological landscape [7].

Notable distinctions in collagen and elastin content are observed between normal and hypospadias-afflicted penises, affecting the stiffness and flexibility of the tunica dartos [7]. There are some proteins that play roles in the elasticity of a tissue. Elastic connective tissue is pivotal in various pathological tissue changes. Elastin, an essential component of the extracellular matrix, endows tissues with extensibility and elasticity. Low elastin concentrations are linked to impaired male urethral development, while elastin-rich concentrations in male fetus urethras elevate intrauterine pressure during the early stages of micturition [14]. Matrix metalloproteinases play a key role in embryological processes and wound healing. MMP-1, a collagenase enzyme, breaks down collagen types I, II, and III, influencing extracellular matrix repair. Decreased MMP-1 levels in hypospadias are linked to increased collagen production. Unbalanced collagen synthesis and degradation can lead to fibrotic tissue formation [15]. Fibroblast growth factors regulate fibroblast growth and differentiation. bFGF stimulates cell proliferation and differentiation, impacting the mesodermal, ectodermal, and endodermal areas. Abnormalities in the bFGF pathway contribute to urethral plate formation, potentially resulting in fibrotic tissue development. α-SMA, implicated in fibrogenesis, drives myofibroblast differentiation and extracellular matrix repair. In the context of hypospadias, α-SMA involvement points to fibrocontractive diseases such as chordee [16].

While this study may provide insights into the differences of the ventral and dorsal tunica dartos, we did not include samples of tunica dartos from healthy patients, which would limit the data analysis only to patients with chordee.

Understanding the differential mRNA expression of fibulin-1, elastin, MMP-1, bFGF, and α-SMA between the ventral and dorsal tunica dartos in patients with hypospadias and chordee presents an opportunity to explore the intricate molecular mechanisms underlying hypospadias development and its associated complications.

## Abbreviations

α-SMA: α-smooth muscle actin
bFGF: basic fibroblast growth factor
Ct: cycle threshold
MMP-1: matrix metalloproteinase-1
mRNA: messenger RNA
qRT-PCR: real-time quantitative reverse transcription–polymerase chain reaction
SPIRIT: Standard Protocol Items: Recommendations for Interventional Trials

## Data Availability

The datasets generated during and/or analyzed during this study are available from the corresponding author on reasonable request
